# Role of the type 3 cytokines IL-17 and IL-22 in modulating metabolic dysfunction-associated steatotic liver disease

**DOI:** 10.3389/fimmu.2024.1437046

**Published:** 2024-08-02

**Authors:** Mohamed N. Abdelnabi, Ghada S. Hassan, Naglaa H. Shoukry

**Affiliations:** ^1^ Centre de Recherche du Centre hospitalier de l’Université de Montréal (CRCHUM), Montréal, QC, Canada; ^2^ Département de microbiologie, infectiologie et immunologie, Faculté de médecine, Université de Montréal, Montréal, QC, Canada; ^3^ Département de médecine, Faculté de médecine, Université de Montréal, Montréal, QC, Canada

**Keywords:** metabolic dysfunction-associated steatotic liver disease, metabolic-dysfunction associated steatohepatitis, liver fibrosis, IL-17, IL-22, sexual dimorphism

## Abstract

Metabolic dysfunction-associated steatotic liver disease (MASLD) comprises a spectrum of liver diseases that span simple steatosis, metabolic dysfunction-associated steatohepatitis (MASH) and fibrosis and may progress to cirrhosis and cancer. The pathogenesis of MASLD is multifactorial and is driven by environmental, genetic, metabolic and immune factors. This review will focus on the role of the type 3 cytokines IL-17 and IL-22 in MASLD pathogenesis and progression. IL-17 and IL-22 are produced by similar adaptive and innate immune cells such as Th17 and innate lymphoid cells, respectively. IL-17-related signaling is upregulated during MASLD resulting in increased chemokines and proinflammatory cytokines in the liver microenvironment, enhanced recruitment of myeloid cells and T cells leading to exacerbation of inflammation and liver disease progression. IL-17 may also act directly by activating hepatic stellate cells resulting in increased fibrosis. In contrast, IL-22 is a pleiotropic cytokine with a dominantly protective signature in MASLD and is currently being tested as a therapeutic strategy. IL-22 also exhibits beneficial metabolic effects and abrogates MASH-related inflammation and fibrosis development via inducing the production of anti-oxidants and anti-apoptotic factors. A sex-dependent effect has been attributed to both cytokines, most importantly to IL-22 in MASLD or related conditions. Altogether, IL-17 and IL-22 are key effectors in MASLD pathogenesis and progression. We will review the role of these two cytokines and cells that produce them in the development of MASLD, their interaction with host factors driving MASLD including sexual dimorphism, and their potential therapeutic benefits.

## Introduction

Non-alcoholic fatty liver disease (NAFLD) is a growing epidemic characterized by fat accumulation in the liver and associated with metabolic disorders and obesity. It affects ~30% of the adult population worldwide with the highest prevalence observed in South America (44.37%) while the lowest rates are seen in Europe (25.10%) (1–3). Consensus from major liver societies have recently coined the umbrella term “steatotic liver disease (SLD)” to include the various etiologies of steatosis (4). NAFLD was renamed metabolic dysfunction-associated steatotic liver disease (MASLD) to account for metabolic dysfunction associated with the development of steatosis. MASLD encompasses a spectrum of liver diseases, ranging from simple fat accumulation or metabolic dysfunction-associated steatotic liver (MASL) to specific pattern of fat accumulation in the liver (hepatic steatosis) and necroinflammatory histologic changes, previously termed non-alcoholic steatohepatitis (NASH) and now metabolic dysfunction-associated steatohepatitis (MASH), and progressing into fibrosis that may eventually lead to complications such as cirrhosis and hepatocellular carcinoma (HCC) (5). Hereinafter, we will use the new nomenclature of MASLD and MASH to refer to NAFLD and NASH, respectively. The progression of MASLD into fibrosis varies among patients, with different degrees of severity and clinical presentations. It is not until recently that the FDA approved a therapeutic agent for the treatment of patients with MASH (6). Resmetirom, a liver-directed thyroid hormone receptor beta–selective agonist demonstrated promising results in a phase 3 trial of patients with MASH progressing to fibrosis (6).

The multifactorial nature of MASLD involves metabolic abnormalities, tissue necrosis, immuno-inflammatory mechanisms, and fibrosis (5, 7, 8). In the context of immune and inflammatory processes, type 3 cytokines are important players in the pathogenesis and/or resolution of liver disease. Namely, IL-17A and IL-22 have significant effector functions during acute liver injury, however their role during the different stages of MASLD remains understudied. In this article, we will provide an overview of MASLD and MASH, and their underlying immune-related mechanisms from host factors to cells. We will focus on the type 3 cytokines IL-17A and IL-22, their roles during the different stages of liver disease in general and MASLD in particular and their therapeutic potential.

## MASLD predisposing conditions

MASLD is promoted by several metabolic and non-metabolic risk factors. Metabolic syndrome (MetS) is considered the strongest metabolic predisposing condition to MASLD, featuring dyslipidemia, obesity, type 2 diabetes mellitus (T2DM), and systemic hypertension (9). Non-metabolic risk factors, including genetic polymorphisms, sex, and age, are also strong influencers of MASLD and its progression. Several genetic variants have been identified in MASLD patients and are reviewed in detail elsewhere (10, 11). Importantly, a specific single nucleotide polymorphism in the patatin-like phospholipase domain-containing protein 3 (*PNPLA3*) gene (rs738409) is strongly correlated with MASLD, disrupting the normal regulation of hepatic triglyceride lipolysis and causing fat accumulation in the liver (12, 13). Sex influences MASLD in the general population and in individuals with MetS, though this link is complex and can be affected by age, MetS, and hepatic inflammation (2, 14). For example, MASLD is more common in men than women, especially before menopausal age (typically ≤ 50-60 years), whereas, after the fifth decade, the prevalence of MASLD becomes similar among men and women or possibly even higher in women (14). Sexual dimorphism holds significant importance in MASLD and warrants particular attention as will be discussed in later sections.

## Animal models of MASLD

The increasing prevalence of MASLD, its association with life-threatening complications and the scarcity of treatment options calls for deeper investigation into its underlying mechanisms using various animal models. Given the multifactorial nature of MASLD, there is no ideal animal model that fully recapitulates the clinical aspects of the disease in humans. Nevertheless, animal models enabled and will further provide mechanistic insights for understanding the disease, and for testing potenital therapeutics (15). Each model has its pros and cons, and the model(s) used in different studies should be considered carefully in data interpretation. Development of MASLD in animal models, mainly mice, could be induced by one of the following triggers: diet, genetic predisposition, or toxins. Different forms of diets (**Table 1**), gene deletions (**Table 2**) or chemical insults (**Table 3**) promote the initiation and progression of MASLD with different degrees of severity or prevalence of a certain feature or phenotype. In some models, a combination of stimuli is needed, for example injecting mice with the chemicals carbon tetrachloride (CCl_4_) or thioacetamide (TAA) together with feeding a high-fat diet accelerates disease progression. **Tables 1**–**3** summarize the most commonly used models in each category and provide a concise description of their main symptoms, advantages and limitations.

**Table 1:** Diet-induced MASLD mouse models.

Diet	Description	Characteristics	Limitations	Refs
**High fat diet (HFD)**	Excess FFAs induces hepatic TGs accumulation and steatosis	•10-50 Wks •Development of metabolic abnormalities and hepatic steatosis	•Mild inflammation and fibrosis development with long-term feeding •MASH development is strain-dependent	(16)
**High fat - High cholesterol diet (HF-HC)**	Higher levels of AST and cholesterol than with HFD.	•12-30 Wks •Development of metabolic abnormalities •Hepatic steatosis after short-term feeding	•Fibrosis development is strain-dependent •MASH appears after 28 Wks post-feeding	(17, 18)
**Methionine and choline deficient diet (MCD)**	Reduced β-oxidation and VLDL production, resulting in hepatic fat accumulation, cell death, oxidative stress and inflammation	•Short duration (2-10 Wks) •Severe MASH and liver fibrosis phenotype •Suitable for rapid testing anti-fibrotic drugs	•Lacks metabolic abnormalities •Not suitable for studying the full spectrum of MASH •Severe weight loss	(17, 19)
**High fat/high cholesterol/high fructose diet** **(also known as AMLN)**	Excessive fructose, a monosaccharide metabolized in the liver, aggravates disease manifestations	•15-30 Wks •Reproducible •Representative of Western diet •Development of metabolic abnormalities and hepatic steatosis •Moderate-severe MASH and fibrosis phenotype	•Certain ingredients like trans fats are replaced by other nutrients •Variable fibrosis	(20)
**Diet-induced animal model of non-alcoholic fatty liver disease (DIAMOND)**	Based on C57BL/6 x S129S1/svlmJ mice fed Western diet (42% fat, 0.1% cholesterol, high fructose/glucose)	•16-52 Wks •Development of metabolic abnormalities •Similar molecular transcriptomic signature to human MASH and MASH-related HCC (except for cholesterol synthesis)	•Strain-dependent •Advanced stages of fibrosis at >6 months, associated with HCC at 12 months post-feeding	(21)

AST, Aspartate Aminotransferase; FFA, Free fatty acids; TGs, Triglycerides; VLDL, very low-density lipoprotein; Wks, Weeks.

**Table 2:** Genetic mouse models of MASLD.

Model	Description	Characteristics	Limitations	Refs
***ob/ob*** **(Leptin deficiency);** ***db/db*** **(Leptin Receptor deficiency)**	Deficiency of leptin or its signaling results in hyperphagia and altered fat distribution in the liver.	•Commercially available model •Development of metabolic abnormalities •Suitable for assessing metabolic risk factors of MASLD	•An additional stimulus such as AMLN or HFD, is needed to induce marked MASH •Variable levels of fibrosis •No leptin mutation exists in human MASH	(15, 22, 23)
***Ldlr^-/-^*** **(LDL receptor deficiency)**	A deficiency in protein involved in endocytosis of cholesterol-rich LDL	•Obesity and hepatic steatosis upon additional challenge with HF-HC diet •A good model to study early MASH onset and fibrosis development given the lipoprotein profile and hepatic inflammation characteristic of MASH	•HF-HC diet induction is required to develop the MASH phenotype	(24, 25)
***Pten^-/-^*** **(deficiency of hepatocyte-specific phosphatase and tensin homolog (PTEN))**	A deficiency of PTEN, a protein with tumor suppressor and metabolic regulating functions	•No additional stimulus is needed to induce MASLD •Histological features simulate human MASH	•No PTEN mutation in human MASH •Lacks metabolic abnormalities •MASH histological features appear at 40 weeks of age	(26–28)

**Table 3:** Chemically-induced MASLD mouse models.

Model	Description	Characteristics	Limitations	Refs
STAM	Injection of low dose of streptozotocin to neonate mice induces cytotoxicity of pancreatic cells followed by additional stimulus at 4Wks of age with HFD	•Short period (8-12 Wks) for inducing fibrosis, and 16 Wks for inducing HCC •Commercially available to assess the progression of MASLD into HCC •Good model of MASH with the diabetes background	•Experimental procedure requires training •Lacks development of obesity	(29, 30)
CCl4 + WD	Injections of a low dose of CCl_4_ along with feeding western diet (WD: 42% fat, 0.1% cholesterol, high fructose/glucose) for 12-24 weeks	•Bridging fibrosis within 12 Wks (not perisinusoidal) •HCC development in 100% of mice •It resembles human MASH at the transcriptomic levels •Suitable for testing anti-fibrotic drugs Suitable for testing anti-fibrotic drugs	•Does not represent a natural cause of human MASH •Metabolic abnormalities are insignificant compared to WD alone •Heterogenous levels of liver fibrosis	(25, 31)
TAA + WD	Injections of low dose of TAA along with feeding western diet (WD: 42% fat, 0.1% cholesterol, high fructose/glucose) for 12-42 weeks	•Severe diffuse fibrosis and hepatic cirrhosis •Suitable for studying MASH-related HCC •Suitable for testing anti-fibrotic drugs	•Does not represent a natural cause of human MASH •Metabolic abnormalities are insignificant compared to WD alone •Heterogenous levels of liver fibrosis	(15, 25, 32)

## Pathophysiology of MASLD

In the past decade, there has been significant progress in understanding the pathogenesis of MASLD. Pathogenic drivers that promote MASLD development are heterogeneous, including various hepatic and extrahepatic factors. For example, insulin resistance, impaired adipose tissue function, lipotoxicity, and the interplay of diverse immune cells, are all pivotal pathogenic factors, that may interact together or in combination with other factors such as gut dysbiosis and genetic predisposition to influence the onset and advancement of MASH (5, 33).

Insulin resistance impairs lipid regulation and enhances accumulation of proinflammatory macrophages in adipose tissues, thus inducing lipolysis and promoting free fatty acids (FFAs) trafficking towards the liver (33, 34). MASL is initiated by disrupted regulation of the FFA metabolism that usually takes place in the normal liver, leading to fat accumulation in this organ (33, 35). An excessive accumulation of hepatic FFA, originating from disrupted adipose tissue, *de novo* lipogenesis, and/or dietary fat intake, exceeds the liver metabolic capacity. This results in lipotoxic metabolite formation and reactive oxygen species (ROS) overproduction inducing hepatocyte death via predominantly apoptosis, and to a lesser extent necroptosis, or pyrotosis (5, 36–38). Consequently, the persistence of hepatocyte death (a hallmark of MASLD progression) induces continuous liver injury, chronic inflammation (MASH), fibrosis progression, and increases the risk of development of HCC (39). Accordingly, understanding this metabolic inflammation and its critical role in the initiation and progression of MASH is crucial for unraveling the underlying mechanisms in MASLD pathogenesis and identifying potential therapeutic targets. Indeed, this inflammatory response is a complex interplay involving numerous innate and adaptive immune cells, each with its unique role. In this section, we briefly discuss the roles of these immune cells and their contributions to the inflammatory aspects of MASH progression.

### Immune cells implicated in MASLD

#### Cells of the innate immune system in MASLD

The infiltration of innate immune cells, mainly neutrophils and macrophages into fatty livers is one of the hallmarks of MASH and highly contributes to disease progression (5, 40). These cells detect damage-associated molecular patterns (DAMPs) and pathogen-associated molecular patterns (PAMPs) from stressed hepatocytes and gut dysbiosis-related bacterial products, respectively (41), and engage in phagocytosis and antigen removal leading to hepatocyte apoptosis and pro-inflammatory cytokine production. Of note, pattern recognition receptors (PRRs) on innate cells are highly increased in MASH, and positively linked to hepatocyte death and disease progression (42, 43).

Liver-resident macrophages, known as Kupffer cells (KCs), play a pivotal role in MASH development through various mechanisms. For example, KCs accelerate hepatic fat accumulation by inhibiting fatty acid oxidation in IL-1α- and TNF-α-dependent mechanisms (44, 45). Recent studies, employing single-cell RNA sequencing technology, have unveiled the heterogeneity and functional diversity of macrophages in MASLD, challenging their conventional dichotomous classification as proinflammatory M1 versus anti-inflammatory/pro-repair M2 subtypes (40, 45, 46). Two distinct subsets of KCs in MASH have been identified based on the expression of the triggering receptor expressed on myeloid cells (TREM)−2. TREM2^low^ KCs were more common in healthy livers, while TREM2^high^ KCs were primarily enriched in MASH livers, and played a protective role against fat accumulation and fibrosis development (47, 48).

Activated neutrophils recruited during liver injury express inflammatory mediators such as myeloperoxidase (MPO) and elastase, and release neutrophil extracellular traps (NETs), all involved in MASH progression (49). MPO, a pro-oxidant enzyme, stimulates the generation of ROS molecules causing hepatocyte death and/or direct activation of hepatic stellate cells (HSCs), and thus enhancing the progression of MASH-related liver fibrosis (50, 51). Neutrophil elastase, a serine protease enzyme, promotes MASH pathology by enhancing the generation of lipotoxic ceramides and upregulation of the inflammatory IL-6 cytokine (52, 53). NET structures which consist of nucleic acids, histones, and antimicrobial peptides, are responsible for containing pathogens and maintaining homeostasis under physiological conditions. NET activation and release, a process known as NETosis is dysregulated in certain pathological contexts, including MASH, mediating detrimental effects such as inducing suppressive regulatory T cell (Treg) differentiation and hence promoting MASH-HCC progression (54). Additional innate immune cells exist in the healthy liver, including dendritic cells (DCs), natural killer (NK), and natural killer T (NKT) cells. They are responsible for various immune functions ranging from maintaining liver tolerance to eliminating antigens of pathogenic origin or malignant nature. However, their role in MASH remains controversial, with opposing functions and divergent effects in liver inflammation and fibrosis, as demonstrated by different studies, highlighting the heterogeneity of these cells and their complex role in MASH (55, 56).

Innate lymphoid cells (ILCs) originate from common lymphoid progenitors (CLPs) but, as innate immune cells, they do not express antigen-specific receptors. Although ILCs are less abundant than other lymphocytes in human and mouse tissues, they are a heterogeneous group. ILCs function similarly to CD4^+^ T helper cells and are considered their innate counterparts. Based on their cytokine profiles, ILCs are classified into three subsets: ILC1, ILC2, and ILC3, corresponding to Th1-, Th2-, and Th17-like profiles, respectively (57). In the liver, ILC1 is the most prevalent subset and is characterized by the expression of the transcription factor T-bet and the production of IFN-γ (57). The role of ILCs in MASLD is not well understood and remains to be fully elucidated. This is likely due to the lack of robust methods to clearly delineate the subsets of ILCs, particularly distinguishing ILC1 from NK cells. Furthermore, in some models deficient in NK cells, there was also a concurrent deficiency in ILCs. Consequently, no clear role for ILCs has been definitively demonstrated (57).

Other innate immune cells are also implicated in MASH pathogenesis. The γδ T is an unconventional T cell type with limited MHC-dependent antigen recognition capacity. In response to pathogens, γδ T cells secrete IL-17A and IFN-γ (58). In a MASH animal model, IL-17A-producing γδ T cells were shown to expand, contributing to enhanced inflammation and liver injury (59). Mucosal-associated invariant T (MAIT) cells, are usually enriched in the healthy liver and involved in its tolerance, they have been understudied with respect to their role in MASH development. These cells could mediate protective effects, specifically at the early stage of MASH, promoting an anti-inflammatory state in macrophages, and hence delaying MASH progression (60). However, at the late stage, MAIT exacerbates MASH-related fibrosis by producing the proinflammatory cytokine IL-17A (61).

#### Cells of the adaptive immune system in MASLD

Growing evidence demonstrated the involvement of adaptive immunity in MASH. This is evident through the formation of focal lymphocyte aggregates, T and B cells, in livers of MASH patients and murine models, contributing to increased lobular inflammation and liver injury (38, 62, 63). Indeed, an enrichment of intrahepatic B cells has been reported in MASH, coinciding with elevated levels of the B cell-activating factor (BAFF) implicated in B cell survival and maturation (62, 64). The role of B cells in MASH involves the release of proinflammatory cytokines that activate both macrophages and HSCs (65, 66). However, these findings are primarily based on *in vivo* animal studies, and their clinical significance remains unclear.

T cells also contribute to MASH pathogenesis. CD8^+^ T cells are enriched in the liver compared to peripheral blood and are the major conventional (CD3^+^) T cell population in the liver (67), in particular, tissue-resident memory CD8^+^ T cells (68, 69). CD8^+^ T cells are involved in maintaining liver tolerance and homeostasis under static conditions (67). In human and murine MASH, there is an expansion of intrahepatic CD8^+^ T cells, primarily driven by IFN-α responses (38, 63, 70). Both pathogenic as well as protective roles have been attributed to CD8^+^ cells in MASH (63, 71), but more research is needed to fully characterize their functions in this context. CD4^+^ T helper cells (Th), include various subsets, i.e. Th1, Th2, Th17 and Tregs, that are capable of mediating different immune responses, with plasticity in their roles based on the signals they receive (72, 73). Th17 cells, responsible for production of IL-17A and IL-22 cytokines, are mainly involved in the protection against infections and in tissue repair (58, 74) while Tregs, producing IL-10 and TGF-β cytokines, maintain immune tolerance and prevent tissue damage during infections (58). A dysregulation of Th17 and Tregs has been reported in MASH and associated with disease development and progression (38, 58). Other helper T cell subsets, their cytokines and functions in health and disease, including MASH, are reviewed elsewhere (58, 75).

In summary, both innate and adaptive immunity contribute to MASH progression and to liver cirrhosis and HCC. Neutrophils and macrophages are key innate drivers, while adaptive immunity likely sustains inflammation and liver fibrosis evolution. Understanding the role of these immune cells is crucial for developing new treatments. In this review, we focus on type 3 immunity and its effector cytokines, IL-17A and IL-22, and their roles in MASLD and MASH-associated fibrosis.

## Type 3 cytokines

Type 3 immunity is a recently introduced term as an additional cell-mediated effector immune response (compared to type 1 and 2) mediated by cells producing the inflammatory cytokines IL-17 and IL-22 (76) and exemplified by IL-17-producing CD4^+^ T cells, known as T helper 17 cells (Th17). Besides Th17 cells, other immune cells are also type 3 cytokine producers, including Tc17, Th22, Tc22, γδ-T cells, MAIT cells, ILC3s, neutrophils, NK-T cells, macrophages and mast cells, under the control of the retinoic acid receptor-related orphan receptor-gamma-t (RORγt) and the aryl hydrocarbon receptor (AhR) transcription factors (77). Type 3 immunity mainly mediates the protection of the epithelial barriers in different tissues, including the liver, against extracellular bacteria and fungi (78). This immunity is also implicated in pathogenic and protective processes in various diseases ranging from inflammatory and autoimmune diseases to cancer (74, 78, 79). There are six members of the IL-17 family (IL-17A-F). Both IL-17A and F have similar biological activities as they share target tissues, cellular sources, and the receptor (IL-17RA/IL-17RC subunits) (80). IL-17B, interacts with the IL-17RB subunit to mediate neutrophil migration, indicating a pro-inflammatory role, and has been associated with poor prognosis in patients with pancreatic and lung cancers (80–82). IL-17C interacts with the IL-17RE subunit, mediating antimicrobial responses and maintaining epithelial barriers in both the skin and intestine (80, 83). However, in psoriatic mouse models, IL-17C exacerbates psoriatic inflammation (84). IL-17D signaling and biological activity remain poorly understood, with limited reports suggesting a proinflammatory effect through the induction of IL-6 and IL-8 (80, 85). Unlike other IL-17 cytokines, IL-17E uniquely promotes a type 2 response involving IL-4, IL-15, and IL-13, which are associated with dampening Th17-driven inflammation observed in autoimmune disorders (86).

Th17 cells also produce IL-22 (discussed in detail in this review) and IL-26, thus all are termed type 3 cytokines. IL-26 is predominantly produced by activated Th1 and Th17 memory CD4^+^ T cells. Specifically, IL-26-producing pro-inflammatory Th17 cells are found in chronically inflamed tissues such as chronic hepatitis C and inflammatory bowel diseases (IBD) (87–89). IL-26 signals through its receptor IL-26R (IL-10R2/IL-20R1 dimer) and can directly kill extracellular bacteria through membrane-pore formation. Additionally, IL-26 activates immune cells, especially plasmacytoid dendritic cells (pDCs), by aiding in the internalization of bacterial DNA from dying bacteria, subsequently triggering a type 1 interferon response via toll-like receptor 9 (TLR9) (87, 90). However, the role of IL-26 in chronic inflammation remains unclear. Finally, Th17 cells also produce type 1 cytokines such as IL-21. IL-21 signals through a heterodimer receptor composed of the IL-21 receptor (IL-21R) and the common cytokine receptor γ-chain (γc) and is involved in the differentiation, expansion, and survival of CD4^+^ and CD8^+^ T cells, as well as B cells (91).

In summary, IL-17A and IL-22 are particularly significant among type 3 cytokines due to the wide investigation of their roles in the initiation, progression, and resolution of liver disease, notably MASLD, and their therapeutic potential and will thus be the focus of this review. IL-17B-F cytokine members, IL-21, and IL-26, are extensively reviewed elsewhere (80, 87, 91).

### The IL-17/IL-17R axis

The most studied members of the IL-17 family of cytokines are IL-17A and IL-17F. These cytokines exhibit 55% homology in their amino acid sequence which is reflected in their comparable functions, sources and targets (92). We will focus on IL-17A, hereinafter referred to as IL-17. Th17 polarization relies on antigenic stimulation, IL-6 and TGF-β, and is stabilized by IL-23 signaling. It is regulated by the activation of several transcription factors including signal transducers and activators of transcription 3 (STAT3), RORγt and AhR, but negatively regulated by Tbet and FOXP3 which promote differentiation of Th1 and Tregs, respectively (78, 93, 94). IL-10-expressing Tregs suppress the differentiation, proliferation, and effector functions of Th17 cells (58, 78). On the other hand, Th17 cells can undergo trans-differentiation to acquire an anti-inflammatory Treg-transcriptional profile, involving the canonical TGF-β signaling and AhR (95).

**Figure 1:**
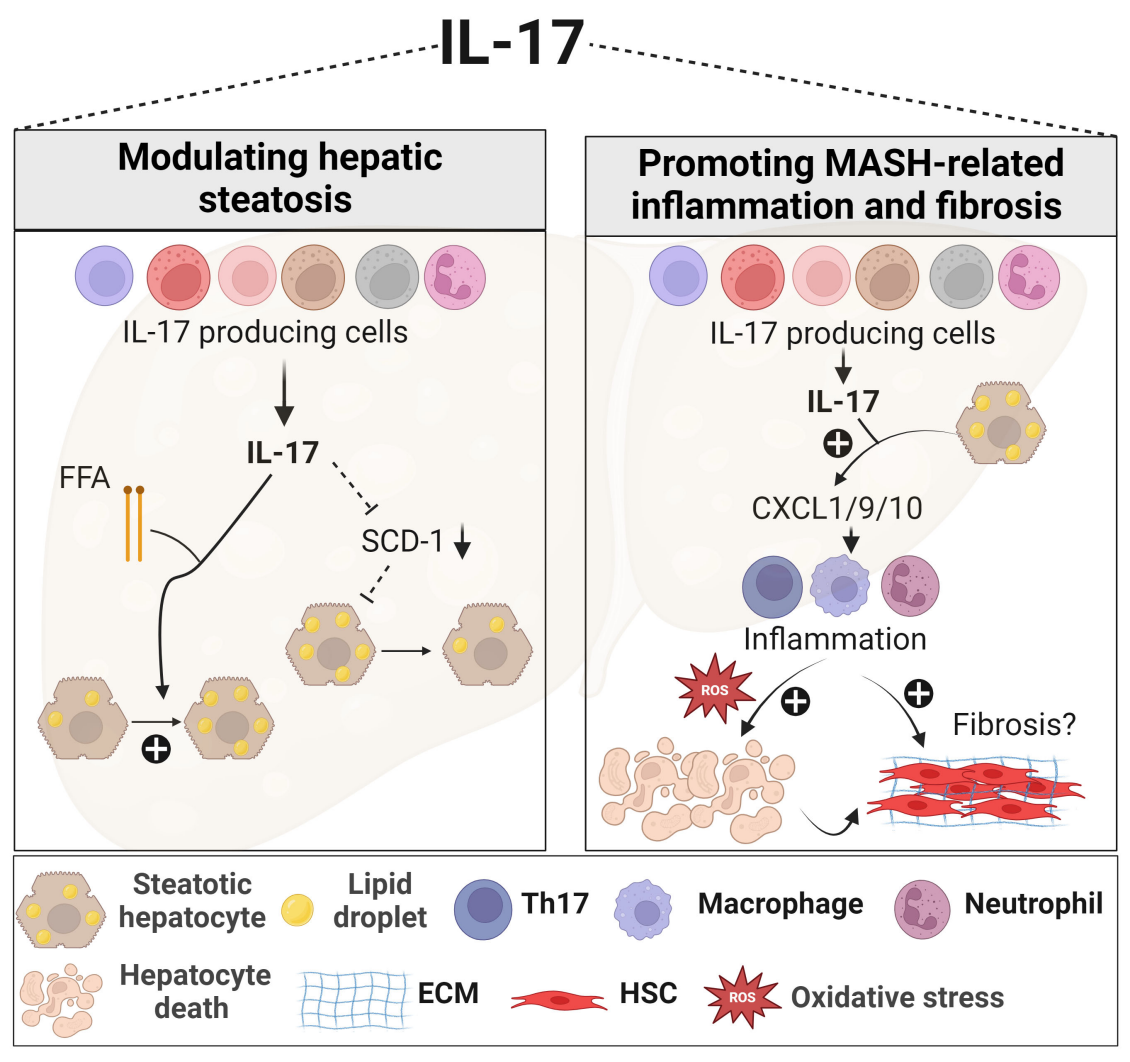
Role of IL-17 in MASLD. In the presence of elevated FFA, IL-17 enhances lipid accumulation within the liver. IL-17 is also associated with reduced hepatic steatosis through downregulating SCD-1, an important enzyme for *de novo* lipogenesis. IL-17 further exacerbates MASH-related inflammation by actively recruiting inflammatory immune cells, including neutrophils, macrophages, and Th17 cells, via promoting hepatocyte release of chemokines such as CXCL1, CXCL9, and CXCL10. These recruited immune cells contribute to oxidative stress, hepatocyte death, and the progression of fibrosis. However, the effect of IL-17 on liver fibrosis remains contradictory. Generated using Biorender.com. ECM, Extracellular matrix; FFA, free fatty acid; HSC, hepatic stellate cell; SCD-1, stearoyl-CoA desaturase-1.

IL-17 signals through the IL-17 receptor, IL-17R, expressed on epithelial cells, fibroblasts, macrophages, and endothelial cells (78, 96). This interaction activates downstream intracellular signaling, including NF-κB, C/EBP, and MAPKs/p38/JNK phosphorylation, as well as a non-canonical pathway that eventually stabilizes mRNA transcripts of target chemokines or cytokines (97, 98). Consequently, the expression of several downstream genes is enhanced, including that of neutrophil chemoattractants (e.g., *CXCL1*) (**Figure 1**), antimicrobial peptides (AMPs), and angiogenic factors (99). Under physiological conditions, these effects of IL-17 protect mucosal surfaces of different organs, including the liver, from external pathogens like bacteria and fungi (76, 78). Numerous clinical and experimental studies have investigated the role of IL-17 in both acute and chronic liver injuries, but several unanswered questions remain.

#### Role of IL-17 in acute and chronic liver injuries

Acute liver injury (ALI) is induced by various insults, such as drugs, toxins, alcohol, and infections and initiates a self-resolving wound-healing response, encompassing three overlapping phases: inflammation, proliferation/repair, and tissue remodeling (78, 100). IL-17 has been implicated in all phases of ALI, mediating different functions. During the inflammation phase, IL-17 in synergy with other cytokines such as TNF-α and IL-6 induces hepatocytes and fibroblasts to release pro-inflammatory chemokines, facilitating the recruitment of monocytes, macrophages, and neutrophils to the injury site (101–104). Genetic deletion or pharmacological blockade of IL-17 ameliorated ALI underscoring the proinflammatory impact of IL-17 in acute hepatitis (101, 105, 106). A hepatoprotective effect of IL-17 in ALI was also reported. Mice with α-galactosylceramide (αGalCer)-induced hepatitis demonstrated an exacerbated inflammation upon neutralization of IL-17. In the hepatitis B virus surface antigen transgenic mouse (HBs-Tg) treated with ConA, depleting γδ T cells, one of the major cellular source of IL-17 aggravated liver damage and pro-inflammatory events (107, 108).

IL-17 also contributes to the tissue repair phase of the wound healing response to injury. IL-17 indirectly enhances the regeneration of hepatocytes and the differentiation of murine liver progenitor cells (LPCs) into hepatocytes by activating macrophages and DCs to produce IL-6 or IL-27 (109, 110). Given that HSCs express the IL-17R, IL-17 can directly activate HSCs promoting their proliferation and increased expression of collagen and alpha-smooth muscle actin (α-SMA) (103, 111), or indirectly via activating other pro-inflammatory cells in the liver microenvironment (101–103, 109). We demonstrated that IL-17 alone does not activate HSCs *in vitro* but sensitizes their response to suboptimal doses of TGF-β by upregulating their expression of the TGF-β receptor, TGF-β-RII (112).

IL-17 could be also involved in the tissue remodeling phase usually characterized by increased expression of matrix metalloproteinases (MMPs) and decreased tissue inhibitor of metalloproteinases (TIMPs) (113). Indeed, IL-17 mediates the expression of MMPs such as MMP-1, - 2 and -9 in inflammatory conditions and cancers (114, 115). Taken together, the role of IL-17 is multifaceted in acute hepatitis, acting as a proinflammatory cytokine in some contexts, while mediating repair in others, and warrants further investigation into its implication in the different phases of ALI.

During chronic liver injury, induced by constant damaging insult and characterized by persistent inflammation and hepatic fibrogenesis (78, 116), the effects of IL-17 have been extensively investigated both in humans and in animal models. The hepatic Th17/IL-17 axis is commonly elevated in patients and mice with chronic liver disease (CLD) and correlates with disease severity (102, 103, 117–119). Indeed, a common process seen in various CLDs is the production of chemokines, such as CXCL9, CXCL10, and CCL20 from damaged hepatocytes that attract Th17 cells expressing CXCR3 and CCR6 receptors (120–122). Other specific triggers of Th17 include hepatitis B virus (HBV) antigens and thymic stromal lymphopoietin from hepatitis C virus (HCV)-infected hepatocytes that stimulate antigen-presenting cells (APCs) to release IL-6 and IL-23, promoting Th17 differentiation (119, 123, 124). In this context, antiviral therapies reduce Th17 response and viral load, resulting in improved liver function in chronic viral hepatitis (CVH) (125, 126). Furthermore, a particular characteristic of CLDs, is an imbalanced Th17/Tregs ratio (127), evident in CVH as well as other CLDs such as alcoholic steatohepatitis (ASH) (102, 128), autoimmune hepatitis (129), and MASH, further discussed below (130–133), exacerbating inflammation and liver damage. Mechanistically, IL-17 promotes inflammation by recruiting and activating myeloid cells (macrophages, and neutrophils), inducing them to produce proinflammatory cytokines, e.g., TNF-α, and exacerbating hepatocyte injury. This was further supported by studies using IL-17 neutralizing antibodies or genetic knockout models, where IL-17 deficiency mitigated inflammation and fibrosis progression by reducing neutrophil infiltration and expression of proinflammatory cytokines (111, 134). In addition to its proinflammatory role, IL-17 can also modulate liver fibrosis progression in CLDs. As previously mentioned, IL-17 can exert a pro-fibrogenic function by acting either directly or indirectly on HSCs, leading to their activation and ECM deposition (103, 111). For example, IL-17 induces fibrosis development by stimulating the proliferation and activation of HSCs and triggering them to release IL-8 and CXCL-1, thus enhancing scar tissue deposition and neutrophil recruitment (102, 135). On the other hand, IL-17 also induces monocytes and KCs to produce pro-inflammatory (e.g., IL-6) and profibrogenic (e.g., TGF-β) cytokines that promote collagen deposition by HSCs (103, 136). Conversely, KCs and HSCs themselves can promote Th17 differentiation through an IL-6-dependent mechanism (137, 138), and IL-17 release via a TLR3-dependent pathway (139, 140), respectively. Pharmacological or genetic inhibition of IL-17 production or its signaling pathway (*Il17a* ^-/-^ or *Il17ra* ^-/-^ mice, respectively) in various liver fibrosis models led to reduced HSC activation and limited liver fibrosis progression (103, 111, 141). Altogether, IL-17 seems to be highly implicated in detrimental inflammatory and fibrogenic effects, contributing to CLD development and progression.

#### Role of IL-17 in MASLD

##### The metabolic effects of IL-17

The Th17/IL-17 axis exerts diverse effects, encompassing metabolic, proinflammatory, and profibrogenic responses in MASLD (**Figure 1**). Regarding the metabolic effects, disrupting the IL-17 pathway through genetic deletion (*Il17a* ^-/-^) or blocking its signaling (*Il17ra* ^-/-^) resulted in increased weight gain and adiposity in models of diet-induced obesity and MASLD (132, 133, 142). Indeed, IL-17 suppresses adipogenesis by downregulating pro-adipogenic transcription factors such as C/EBP-α and PPARγ (143, 144). A protective effect for IL-17 in the development of hepatic steatosis in MASLD was reported. The lack of IL-17 in various MASLD murine models was associated with increased fat accumulation in the liver (**Figure 1**), affecting mainly the expression of stearoyl-CoA desaturase-1 (SCD-1), a regulator of lipogenesis (132, 145–147).

On the other hand, IL-17 promotes insulin resistance and hinders glucose uptake in adipose tissue and the liver (132, 133, 148). Furthermore, stimulating hepatocytes with IL-17 *in vitro*, in the presence of FFAs, upregulated their lipid content (**Figure 1**), via inhibition of insulin-signaling pathways, and induced IL-6 production (131, 133). This latter IL-17-mediated effect together with the known role of IL-6, in synergy with TGF-β in enhancing the differentiation and proliferation of Th17 (149), constitute a positive feedback loop between hepatocytes and Th17 cells in the context of MASLD. Similarly, Th17 cells sustain adipose tissue inflammation by promoting IL-6 and IL-1β secretion from adipocytes, monocytes, and macrophages (133, 144, 150).

Despite these paradoxical metabolic effects, the inhibitory function of IL-17 against adipogenesis suggests a regulatory potential of this cytokine to control obesity and limit excess adiposity in MASLD. Nevertheless, chronic low-grade inflammation associated with MASLD, driven by IL-6 or IL-1β cytokines released from adipose tissue macrophages, could counteract these protective effects of IL-17 (151). Further research is essential to elucidate the precise role of IL-17 in MASLD-related hepatic steatosis.

##### The inflammatory effects of IL-17

The role of IL-17 in inducing MASH-related inflammation and promoting liver injury is well established. Hepatic Th17 or IL-17^+^ cells and Th17-related genes, including RORγt and IL-23, are enriched in MASH patients (130, 131, 133, 141, 152). Notably, a distinctive Th17 subset, termed inflammatory hepatic CXCR3^+^ IL-17^+^ IFN-γ^+^ TNF-α^+^ Th17 (ihTh17) was detected in the livers of MASLD patients. This subset exhibited a higher inflammatory profile compared to conventional hepatic CXCR3^-^Th17 (chTh17) cells and correlated with MASLD severity (152). Moreover, genome-wide association studies (GWAS) revealed single nucleotide polymorphisms (SNPs) in key IL-17 axis pathways, including RORγt, STAT4, and IL-17R that were associated with the severity of human MASLD and hepatobiliary disease (153, 154). Moreover, in obese MASLD patients, an elevated Th17/Treg ratio in the peripheral blood and liver was positively linked to the transition from MASL to MASH, highlighting Th17 involvement at the initiation and the progression of MASH-related inflammation (130). Importantly, the ratio of Th17 to resting Tregs, but not activated Tregs, distinguished MASL from MASH patients and correlated with the hepatocyte death marker cytokeratin18 (130). In the same study, one-year post-bariatric surgery, the hepatic Th17/Treg ratio significantly reversed to normal levels and associated with improved MASH. In line with this, the peripheral and hepatic venous IL-10/IL-17 ratio was markedly reduced in morbidly obese MASH patients, signifying an inflammatory state compared to those without MASH (155).

As described in humans, upregulation of the IL-17 axis and an imbalance of the Th17/Tregs ratio were observed in several murine MASH models and linked to adverse outcomes of the disease (131–133, 146, 147, 152, 156). Interventions targeting IL-17 or its signaling through pharmacological inhibitors, neutralizing antibodies or genetic deletion (*Il17a* ^-/-^ or *Il17ra* ^-/-^), effectively decreased inflammation in several MASH models by reducing hepatocyte injury, the infiltration of pro-inflammatory immune cells, and the release of chemokines and cytokines (131–133, 145–147, 156). Despite the uncertainty as to the mechanisms underlying IL-17 detrimental effects in MASH, some potential pathways have been suggested. In methyl choline-deficient diet (MCD)- or high fat diet (HFD)-induced MASH models, the Th17/IL-17 axis increased hepatic CXCL-10 via an NF-κB/p65-dependent mechanism (**Figure 1**), promoting the recruitment of inflammatory macrophages and T cells (145, 146). Furthermore, the upregulated CXCR3-CXCL9/10 axis in the fatty liver environment, attracts ihTh17 cells that then induce macrophage infiltration and hepatocyte ballooning and thus exacerbate MASH progression (**Figure 1**) (152). Another mechanism that underscores the implication of Th17/IL-17 in MASLD-related inflammation, is its capacity to induce ROS production in a NADPH oxidase-dependent manner (132). Other functions of IL-17 including induction of IL-6 release from hepatocytes in the presence of FFA, further contribute to its inflammatory signature and role in MASLD (131). As to Tregs, some studies reported that their numbers were decreased in MASLD models (157) while others reported that their levels were unchanged (147, 158) or even increased (159, 160). Mechanisms underlying these contradicting observations were not well explored. A deeper investigation into the role of Tregs and their proportion as compared to Th17 cells, is needed in the context of human and mouse models of MASH.

##### The fibrogenic effects of IL-17

Like other CLDs, the Th17/IL-17 axis contributes to MASH-related fibrosis by enhancing inflammation, hepatocellular injury, and the activation of HSCs (**Figure 1**). However, evidence supporting the profibrogenic role of IL-17 in MASH is primarily derived from a small number of pre-clinical studies. IL-17 deficiency (*Il17a* ^-/-^) in the MCD-induced models was shown to reduce liver fibrosis (147), while treating the HFD-induced MASH model with recombinant IL-17 worsened fibrosis development (133). However, divergent findings were observed in another MCD-fed IL-17-deficient model, where similar level of liver fibrosis was detected when compared to wild-type animals (145). Taken together, further research is needed to clarify the fibrogenic role of IL-17 and its modulation of HSCs in MASH.

#### IL-17 as a therapeutic target in MASLD

Targeting the IL-17 axis is a promising therapeutic intervention to manage the onset and progression of MASLD (161). Direct inhibition of IL-17 through monoclonal antibodies (e.g., secukinumab and ixekizumab) or of IL-17R, (brodalumab) has been approved for treating autoimmune diseases like psoriasis, psoriatic arthritis, and ankylosing spondylitis (161, 162). Indirect inhibition of IL-17 that involves blocking Th17 cell generation and related cytokines was also evaluated for therapeutic potential. Blocking the IL-6 (e.g., tocilizumab), IL-1β (e.g., canakinumab), or IL-23 (e.g., guselkumab) pathways partially reduces Th17 cell differentiation or survival (163–166). Additionally, targeting RORγt/RORc with small molecule antagonists (e.g., S18-000003) or inverse agonists (e.g., SHR168442) restores the Th17/Tregs balance and suppresses Th17-related cytokine production (167). Inhibiting the IL-17 pathway itself has shown promising outcomes in preclinical ASH studies. For example, IL-17 exacerbated alcohol-induced liver/brain axis injury, highlighting its involvement in systemic effects and potential contribution to brain disorders (168). Interestingly, using an anti-IL-17 antibody or an RORγt inhibitor not only reversed alcohol-induced injury of the liver/brain axis in mice with severe ASH but also reduced their voluntary alcohol drinking (168). In an experimental ASH model, genetic ablation of the IL-17RA in steatotic hepatocytes downregulated TNFRI, sterol regulatory element-binding proteins 1/2 (SREBP1/2), and 7-dehydrocholesterol reductase (DHCR7), leading to protection against hepatic steatosis and HCC development (169). In the CCl_4_-induced chronic liver injury, we observed reduced levels of intrahepatic IL-17^+^ cells including neutrophils, upon inhibiting RORγt with GSK805 resulting in decreased liver fibrosis (141). Nevertheless, no clinical studies have investigated the efficacy of IL-17 inhibitors in the context of MASLD.

It is important to note, that blocking the IL-17 pathway is linked with adverse outcomes such as injection site reactions and upper respiratory infections given the role of the IL-17 axis in mucosal immunity against bacterial and fungal infections (161). Moreover, patients with Crohn’s disease showed no amelioration or even reactivation of their disease upon treatment with anti-IL-17 drugs (170, 171). Furthermore, inhibiting RORγt has been associated with altered T cell receptor (TCR) α gene rearrangement, reducing T cell diversity and increasing the risk of thymic aberrations (172, 173). Thus, the use of inhibitors of the IL-17 pathway in treating CLDs, including MASLD, still needs further assessment to establish the balance between effectiveness and safety.

### The IL-22/IL-22RA1 axis

IL-22, initially known as IL-10-related T cell-derived inducible factor (IL-TIF), belongs to the IL-10 cytokine family (174). Various adaptive and innate immune cells produce IL-22 such as Th17, Th22, CD8^+^ T (Tc22), γδT, NKT cells, ILC3s, neutrophils, and macrophages (175). IL-22 interacts with its receptor (IL-22R), a heterodimeric receptor composed of IL-22RA1 and IL-10RB2 subunits (176, 177). Notably, IL-22RA1 is predominantly expressed on epithelial cells, but also on fibroblasts and LPCs (178). This unique characteristic highlights the novelty of IL-22 as a cytokine affecting mainly epithelial cells. IL-22 binds to the IL-22R and triggers downstream signaling primarily through STAT3 activation but also via STAT1 and STAT5 pathways (179). IL-22/IL-22R induces phosphorylation of Janus kinase 1 (JAK1) and tyrosine kinase 2 (TYK2) that allow the translocation of STAT3 into the nucleus (175, 179). In addition to the JAK-STAT3 axis, IL-22 downstream signaling also involves activation of MAPK, PI3K, and AKT-induced mTOR pathways (179, 180). Consequently, all these pathways play a crucial role in regulating genes essential for innate immune defense, including the production of AMPs, acute phase proteins, proinflammatory mediators, and tissue regeneration (78, 175).

IL-22 induces the expression of several AMPs like mucus-associated proteins (MUC1), S100A7/8/9, regenerating islet-derived protein family (REG3β), and lipocalin 2 (LCN2) across various epithelial tissues including the liver (181–184). The antimicrobial function of IL-22 is underscored *in vivo* as it restricts the replication and dissemination of *Klebsiella pneumoniae* in the liver and lungs of infected mice (181, 185). Moreover, IL-22 signaling promotes the survival of hepatocytes, by inducing the expression of anti-apoptotic molecules like B-cell lymphoma 2 (BCL-2) and B-cell lymphoma-extra-large (BCL-XL) and thus protecting against hepatitis (186–188). In addition, IL-22 elevates acute-phase proteins such as CXCL-1, serum amyloid A (SAA), and LPS-binding proteins in hepatocytes (189). Interestingly, despite the restriction of IL-22RA1 to epithelial cells, IL-22 can indirectly influence the recruitment of immune cells. In the skin and lungs, IL-22 suppresses the recruitment of Th17 or Th2 cells through CCL17 and CCL22 chemokines, respectively (181, 190). In addition, it was shown that the proliferation of LPCs and intestinal stem cells (ISCs) is enhanced through the IL-22-induced STAT3 mechanism, facilitating tissue regeneration in both acute and chronic injuries (191–193). The influence of IL-22 on HSCs is controversial, with reports suggesting either inhibitory or activating effects, as discussed further below (194–196). Altogether, these conflicting roles for IL-22 warrant further investigation into its implication in the physiology and pathology of different anatomical sites.

### Regulation of IL-22: Focus on IL-22BP

Several cytokines or transcription factors can either positively or negatively regulate IL-22 expression. IL-23 and IL-1β, primarily produced by DCs and macrophages, are key stimulators of IL-22 production from ILC3s, Th17, γδ T, and NKT cells (175, 197–199). Conversely, TGF-β can directly inhibit IL-22 expression through the c-maf transcription factor or indirectly by inhibiting Th17 differentiation and promoting Treg polarization (197, 200, 201). Additional cytokines like IL-25, IL-27, and IL-38 can inhibit IL-22 production, although their precise mechanisms remain unclear (175). The transcription factors AhR, RORγt, STAT3, and Notch are critical regulators of IL-22 expression. Specifically, AhR, activated by ligands from the diet or microbiome, can directly upregulate IL-22 and influence Th17 and ILC3s development (202, 203).

IL-22 binding protein (IL-22BP) or IL-22RA2 acts as a decoy receptor that modulates IL-22 activity, by specifically blocking IL-22 interaction with IL-22RA1. Its high affinity for IL-22 binding (1000-fold greater than IL-22RA1) suggests tight regulation of the activity of this cytokine (204, 205). IL-22BP is expressed in various epithelia, including the liver, with immune cells like DCs, eosinophils, and CD4^+^ T cells as its main producers (175, 206, 207). Of note, the production of IL-22BP itself is influenced by various factors. For example, in an inflamed mouse colon, activation of the inflammasome likely inhibits IL-22BP expression by inducing IL-18 (208), while retinoic acid in DCs, enhances IL-22BP expression (209). In the healthy gastrointestinal (GI) tract, IL-22BP regulates IL-22 activity in the follicle-associated epithelium, impacting mucin levels, AMPs production, and proliferation of epithelial cells (208, 210). Although the absence of IL-22BP alters these mucosal effects, its influence on the microbiome is minimal (210). In contrast, mice lacking IL-22 developed dysbiosis in their GI microbiome (211). This suggests that high levels of IL-22, in the absence of IL-22BP, do not influence microbiome composition, while deficiency of IL-22 has a significant impact on mucosal immunity. Interestingly, despite the potent neutralizing activity of IL-22BP, its expression levels, particularly in the GI tract, do not often parallel IL-22 levels. IL-22BP levels are high during homeostasis, though they are downregulated during inflammation. For example, in colitis models or following LPS administration, IL-22 levels significantly increase, while IL-22BP drops (212). However, a prolonged IL-22 rise can subsequently induce IL-22BP elevation, neutralizing IL-22 activity. This suggests that IL-22BP could mediate IL-22 signaling after the initial effects of this latter have been established, ensuring adequate mucosal immunity and tissue regeneration, and preventing detrimental IL-22 effects (208, 212). Nevertheless, in the context of colorectal cancer, the lack of IL-22BP *in vivo* is linked to disrupted IL-22 signaling, impaired wound healing, and an elevated risk of tumor development (208). The role of IL-22BP/IL-22 axis in liver injury and MASLD is discussed later in this review.

### Role of IL-22 in acute and chronic liver injuries

IL-22 is implicated in the three phases of the wound-healing response following an acute insult. Consistent findings from diverse experimental models, including both infectious and non-infectious causes of ALI, converge on IL-22 exerting a hepatoprotective effect during the inflammatory phase (186, 187, 213–216). Specifically, IL-22 induces the expression of glutathione and anti-apoptotic molecules (BCL2, BCL-XL) in hepatocytes, thereby inhibiting hepatocyte death and mitigating oxidative stress (186–188, 214, 215). Other evidence suggests that the ability of IL-22 to reduce hepatocyte apoptosis is likely mediated by activating autophagy, particularly through the involvement of autophagy-related gene (ATG)4 and ATG7 proteins (217). In the proliferation/regeneration phase, IL-22 facilitates the proliferation of both hepatocytes and liver LPCs by inducing the expression of cyclin D and c-myc (186, 187, 193, 214). The implication of IL-22 in the tissue remodeling phase of ALI is not yet fully understood. Indeed, the effect of IL-22 on HSCs and hence the ECM is controversial, with reports suggesting inhibition of HSCs proliferation (194), enhancement of their activation (141, 195), or promotion of their senescence by IL-22 (218). Despite these hepatoprotective effects of IL-22 in ALI, several studies have shown contrasting findings, highlighting a rather pathogenic role for IL-22. In a rat allogeneic liver transplantation model, IL-22 exhibited hepatoprotective effects during the ischemia-reperfusion injury stage (day one), though it appeared to be pathogenic during the acute rejection stage (day 7), exacerbating liver injury (216). This detrimental effect of IL-22 was attributed to the upregulation of pro-inflammatory cytokines and chemokines, leading to an imbalance in Th17/Tregs (216). Consistently, in ischemia-reperfusion and APAP-induced liver injury models using IL-22BP-deficient mice (*Il22ra2* ^-/-^), the dysregulated IL-22 signaling, marked by upregulated CXCL-10 expression, resulted in increased hepatic inflammatory monocytes and hepatocyte death, exacerbating liver injury (219). In line with the pathogenic signature of IL-22, blocking this cytokine using a neutralizing anti-IL-22 antibody markedly decreased the recruitment of pro-inflammatory immune cells in HBV-transgenic T-cell adoptive transfer model, mitigating ALI (220). Alternatively, the presence of other inflammatory cytokines, such as IL-17, could contribute to the detrimental effects of IL-22 (103, 221). This generally suggests opposite effects of IL-22 rather than a paradox, which is context-dependent and may rely on the specific inflammatory response, the time of exposure, IL-22BP regulation, and the activated signaling pathways in target cells (pro-survival/regeneration vs. pro-inflammatory) (103, 204, 216, 218).

The dual nature of IL-22 functions has also been reported in CLDs. Intrahepatic enrichment of IL-22 in chronic hepatitis B (CHB) or C (CHC) patients, while not directly impacting the replication of HBV and HCV in hepatocytes (120, 214, 220, 222, 223), suggests a crucial role of the cytokine in the progression of liver disease. These elevated IL-22 levels during CHB and CHC infections could contribute to chronic inflammation and increased severity of liver fibrosis (120, 141, 195, 214), by enhancing the recruitment of proinflammatory Th17 cells into the liver and a persistent activation and proliferation of HSCs (120, 195). On the other hand, other studies reported a negative association between intrahepatic IL-22 levels and liver inflammation as well as fibrosis in CHB patients (193, 224), probably due to the capacity of IL-22 to promote the survival of hepatocytes and induce the proliferation of LPCs (224). Similarly in ASH patients, increased levels of circulating IL-22-producing CD4^+^ T cells, including Th17 cells is associated with favorable short-term prognosis (225). Experimental evidence also supports a protective function for IL-22 against ethanol-induced chronic liver injury in mice (226, 227). In this model, reduced IL-22 production by gut ILC3s contributed to an impaired regenerating islet-derived protein 3 gamma (REG3G) and enhanced gut dysbiosis (228) while the administration of IL-22-producing engineered bacteria or recombinant IL-22 alleviated gut and hepatic symptoms (226, 228).

The role of IL-22 in liver fibrosis appears even more complex and context-dependent, with conflicting findings reported in various studies. An anti-fibrotic role has been attributed for IL-22 via its effect on HSCs, inhibiting their activation or inducing their senescence in a STAT3-dependent mechanism. These results were mainly observed in models with supraphysiological expression of IL-22 (IL-22 transgenic (Tg) mice or upon treatment with recombinant IL-22) (103, 194, 218, 229). In line with the anti-fibrotic function of IL-22, patients with *Schistosoma japonicum* infection had elevated hepatic IL-22 and decreased IL-22BP transcripts, correlating with reduced fibrosis and portal hypertension (230). On the other hand, other evidence demonstrated rather a pro-fibrogenic function for IL-22 in CLDs. Specifically, upon an *in vitro* stimulation with IL-22, HSCs exhibited reduced senescence and increased proliferation (195), and showed enhanced sensitivity to TGF-β signaling (141). This fibrogenic effect was confirmed in the chronic CCl_4_ model, where mice lacking IL-22 signaling (*Il22ra1^-/-^*) exhibited reduced hepatic fibrosis compared to their wild-type counterparts (141). These different results as to the role of IL-22 in CLDs might be due to the variation in the animal model employed (IL-22 Tg vs *Il22ra1^-/-^*) and to exogenous versus endogenous IL-22. Nevertheless, uncertainty remains regarding whether circulating IL-22 levels accurately mirror hepatic levels of the cytokine in these individuals (231). In summary, despite variations between data from HSC cell lines and *in vivo* models, the dual role of IL-22 points toward a context-dependent effect regulated at multiple levels.

### Role of IL-22 in MASLD

#### The metabolic effects of IL-22

**Figure 2:**
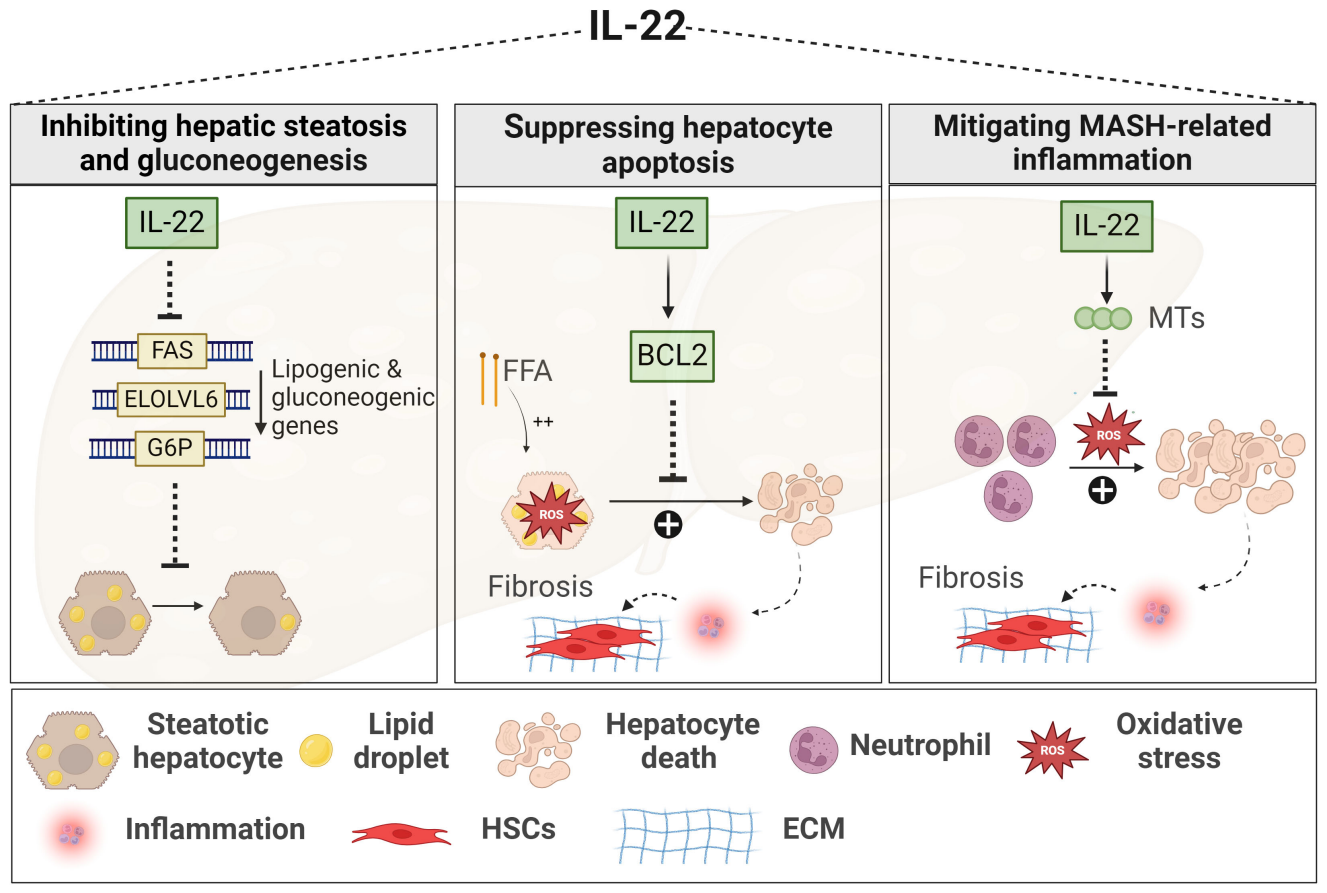
Role of IL-22 in MASLD. IL-22 downregulates the expression of lipogenic genes, such as FAS, ELOLV6, and G6P, thereby limiting lipid accumulation within the liver and reducing hepatic steatosis. IL-22 promotes hepatocyte survival against oxidative stress induced by either FFA or inflammatory neutrophils. This hepatoprotective mechanism involves the induction of anti-apoptotic signals, such as BCL2, and anti-oxidant signals, such as MTs, leading to decreased MASH-related inflammation and fibrosis. Generated using Biorender.com. BCL2, B-cell lymphoma 2; ECM, Extracellular matrix; ELOLV6, elongation of long-chain fatty acid family member 6; FAS, fatty acid synthase; FFA, free fatty acid; G6P, gluconeogenic genes like glucose 6 phosphate; HSC, hepatic stellate cell; MTs, metallothioneins.

The protective effects of IL-22 in MASLD fall mainly under its beneficial functions against MetS and liver injury events (**Figure 2**). In various models of diet-induced MASLD, IL-22 improved glucose tolerance, enhanced insulin sensitivity, and reduced body weight along with decreased adiposity (147, 232–234), via mechanisms dependent on STAT3 and Akt activation (232–234). In addition, IL-22 was shown to inhibit several hepatic lipogenic genes (**Figure 2**) (e.g., fatty acid synthase (FAS)), thereby reducing liver steatosis as well (234–236). These protective metabolic effects of IL-22 were demonstrated upon administration of exogenous IL-22 or in IL-22 Tg mice, rather than being associated with endogenous levels of the cytokine which are usually low in circulation and livers of HFD-induced MASLD (232, 233, 237).

#### The inflammatory and fibrogenic effects of IL-22

IL-22 also exerts protective functions in the context of MASH (147, 238, 239). A human recombinant IL-22 fusion protein, IL-22Fc remarkably ameliorated liver inflammation and fibrosis in neutrophil-driven MASH by suppressing oxidative stress via STAT-3-mediated induction of the antioxidant metallothionein proteins (**Figure 2**) (MT1 and MT2) (238). In line with this, liver-targeted delivery of the IL-22 gene in an HFD-induced MASH model promoted hepatocyte survival and proliferation (**Figure 2**) (234). Again here, these effects of IL-22 were observed upon its exogenous uptake (234, 238). Nevertheless, some studies still demonstrated a protective role for endogenous IL-22 against liver inflammation and fibrosis, particularly in MCD-induced MASH, but such effect was contingent on the absence of the IL-17 cytokine (*Il17a* ^-/-^) (147). Another study reported that elevated levels of IL-22-producing ILC3s in the liver of HFD-induced MASH model were associated with reduced hepatic steatosis, while genetic deficiency of ILC3s worsened liver inflammation and fibrosis progression (**Figure 2**) (239). Of note, IL-22BP and its regulation of IL-22 activity were not examined in these experimental MASLD studies. In summary, cumulative evidence from *in vivo* MASLD models suggests multiple beneficial effects of IL-22, providing protection not only against MetS but also against liver damage and fibrosis progression. Yet, limitations in the experimental studies, as mentioned above, and the absence of clinical investigations hinder a conclusive determination of the beneficial role of IL-22 in the context of MASH.

#### The Therapeutic Potential of IL-22 in MASLD

Phase 1 clinical trials employing IL-22Fc reported good tolerability and safety in healthy human subjects (240, 241). This was associated, even at significantly increased serum IL-22 levels (up to 2000 ng/ml), with induced acute-phase proteins and minimal side effects (240, 241). Importantly, IL-22Fc reduced inflammatory markers and improved clinical scores in a phase 2a open-label trial for moderate to severe ASH (242). Also, in another phase 2 study, IL-22Fc, when combined with systemic corticosteroids, demonstrated safety and efficacy in treating lower gastrointestinal acute graft-versus-host disease (GVHD) (243). The treatment was associated with a distinct fecal microbiota composition, indicating an improvement in GVHD-associated dysbiosis (243). These studies highlight a potential pharmacological advantage of IL-22Fc in both healthy and diseased subjects with minimal adverse events.

Additional evidence supported a mitochondria-protecting function of IL-22Fc against liver injuries induced by HFD or acetaminophen (244, 245). Mechanistically, this was dependent on STAT-3-mediated activation of mTOR and AKT and/or STAT-3-activating AMPK pathways, leading to decreased ROS production, and preserving mitochondrial integrity (244, 245). Moreover, IL-22Fc demonstrated protective effects in acute-on-chronic liver failure (ACLF), a high-mortality syndrome marked by acute decompensated cirrhosis and intense systemic inflammation (246). In an ACLF model, involving chronic CCl_4_ treatment, an acute high CCl_4_ dose and an infection with *Klebsiella pneumonia*, IL-22Fc was capable of reversing the anti-regenerative IFN-γ/STAT1 cascade to the pro-regenerative IL-6/STAT3 pathway. This resulted in reduced bacterial load, enhanced anti-apoptotic signals in hepatocytes, and improved survival rates in mice (246). In contrast, another study demonstrated that progression of ACLF and high mortality rates in diseased patients correlated with elevated serum levels of endogenous IL-22 and a low IL-22BP/IL-22 ratio (247). Nevertheless, this discrepancy in observations may be attributed to the assessment of endogenous versus exogenous IL-22 or of systemic versus hepatic functions of IL-22. Overall, despite promising outcomes indicating the potential efficacy of IL-22 therapy in mitigating MASLD progression, concerns exist regarding its application in CLDs due to its potential to promote inflammation in certain contexts and the risk of adverse off-target effects. Therefore, further clinical studies are needed to thoroughly assess the efficacy and safety of IL-22 therapy in MASLD.

## Sexual dimorphism in MASLD

Sexual dimorphism influences immune responses to infections and inflammatory diseases. Females generally exhibit stronger innate (e.g., phagocytic activity of neutrophils) and adaptive (e.g., CD4^+^/CD8^+^ T cell ratio) immunity than males (248–250). These sex-specific differences comprise a network of interconnected factors like reproductive hormones, genetics, epigenetics, and environmental elements. Yet, these factors have not been fully characterized to elucidate the molecular mechanisms underlying sexual dimorphism in the liver during health and disease (251). In this section, we will focus on sex hormones, specifically testosterone as an androgen for males and estradiol as an estrogen for females. Other factors are reviewed in depth elsewhere (248, 250).

### Sex hormones-related inflammation and fibrosis in MASLDs

The liver itself as well as various innate and adaptive immune cells are major targets for sex hormones as they express androgen (AR) and estrogen receptors (ER). Thus, sex hormones play a fundamental role in MASLDs and CLDs in general, since they modulate liver functions and immune responses (250, 252). Of note, advanced MASH-related fibrosis and/or HCC are generally more prevalent in males than females (253, 254), while autoimmune hepatitis and ASH progress more aggressively in females compared to males (255). Although this could imply that female sex hormones may not often provide resistance against liver injury, it suggests a protective effect in the context of MASH, especially at the premenopausal age.

Experimental studies with estrogen-deficient mice or administration of estradiol into diet-induced obesity (DIO) models revealed that estradiol protects against hepatic steatosis by inhibiting *de novo* lipogenesis, enhancing free fatty acid (FFA) oxidation, and influencing triglyceride and cholesterol metabolism (256–258). Although these protective effects are more pronounced in livers of females, the conversion of testosterone to estradiol in male livers has also been observed, but to a lesser extent, and correlated with improving hepatic insulin sensitivity and bile acid synthesis (259). In line with this, low testosterone levels in males with MASLD were linked to MetS and hepatic steatosis (260, 261). Other studies reported that male mice lacking androgens (specifically testosterone) or AR to have favorable effects against their hepatic steatosis upon testosterone replacement (251, 262). In contrast, females with polycystic ovary syndrome (PCOS), characterized by excessive androgen production, had increased hepatic steatosis, overweight, and MetS, rendering them more susceptible to developing MASLD (263, 264).

Estradiol exerts an anti-lipogenic influence and multiple *in vivo* studies also reported an anti-inflammatory implication of this hormone in MASLD models. With respect to the anti-inflammatory role of estradiol, it has been demonstrated that ovariectomized female mice on HFD exhibit increased levels of hepatic macrophage infiltration, along with pro-inflammatory cytokines (IL-1β, IL-6) and chemokine (CCR2), compared to controls (sham-operated mice) (265, 266). Moreover, IL-6 production is suppressed in KCs upon estradiol administration in male mice harboring DEN-induced HCC (267). Estradiol enhanced apoptosis in activated HSCs, suppressing MAPK pathways (ERK and p38) and protecting against liver fibrosis induced by chemicals like CCl_4_ and thioacetamide in female mice (268–270). Similarly, ovariectomized female mice on a high-glucose and HFD developed significant collagen fibril accumulation as compared to control sham-operated females on the same diet (266). Mechanisms underlying this anti-fibrotic role of estradiol in MASH remain however undefined.

In summary, sex hormones, specifically estradiol, may offer therapeutic advantages in mitigating hepatic steatosis and MASLD-related inflammation/fibrosis in both sexes. Nevertheless, the use of hormonal therapy as a hepatoprotective drug hasn’t been conclusively established, and more studies are needed to explore the impact of sex hormones on immune cells in the liver, further defining their role in MASLD and ultimately identifying potential therapeutic targets for personalized medicine.

### Sex-based differences of IL-22 and IL-17 in MASLD

**Figure 3:**
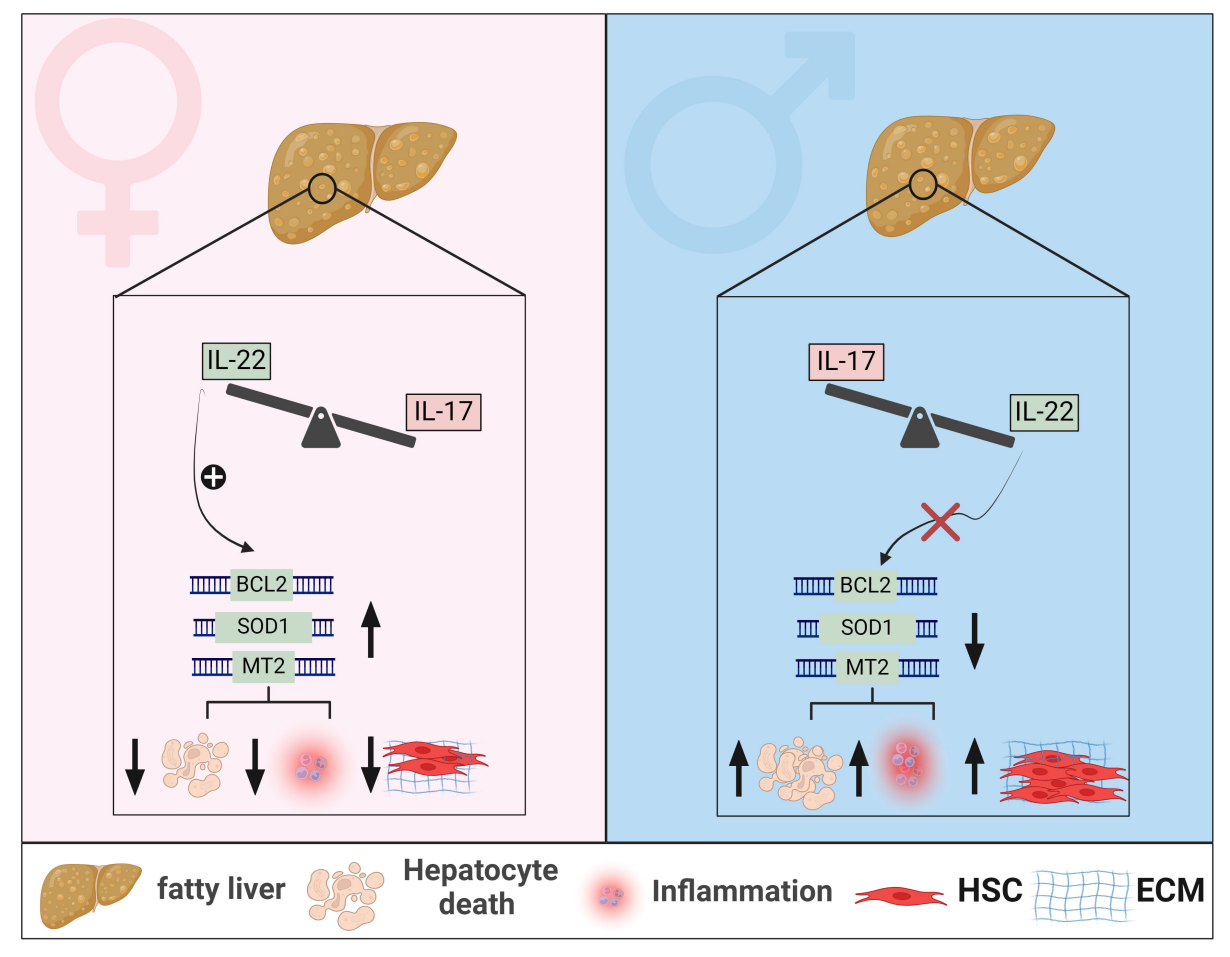
Sexual dimorphism of IL-22 and IL-17 effects in MASLD. In livers of female mice with MASLD, IL-22-producing cells are elevated, whereas in male mice with MASLD, cells producing IL-17 are predominantly enriched. Sexual dimorphism of hepatic IL-22 is associated with increased hepatic expression of anti-apoptotic genes, such as BCL2, and anti-oxidant genes such as SOD1 and MT2, in female mice, leading to mitigated hepatocyte death, reduced MASH-related inflammation, and decreased fibrosis progression. These hepatoprotective effects of IL-22 are absent in livers of male mice with MASLD. Generated using Biorender.com. BCL2, B-cell lymphoma 2; ECM, Extracellular matrix; HSC, hepatic stellate cell; MT2, metallothionein 2; SOD1, Superoxide dismutase 1.

Earlier studies of MASLD have focused primarily on using male mice given the higher prevalence of the disease in males. However, given the important roles that IL-17 and IL-22 play in MASLD and with the increased awareness of the key role of sex-dependent differences observed at the level of these cytokines and progression of MASLD, recent studies integrated males and female to better investigate the contribution of sexual dimorphism to MASLD, and more specifically with respect to the IL-17/IL-22 levels and functions. These studies are summarized in this section and **Figure 3**.

### Sex-related differences in the expression and function of IL-22 and IL-17

The sexual dimorphism-related signature of IL-17 in liver diseases has been demonstrated in the context of autoimmune conditions. For example, in an acute cholangitis model, females developed pronounced cholangitis, driven by increased recruitment of endogenous Th17, along with elevated expression of the chemokines CXCL-9 and CXCL-10 (271). Testosterone treatment of these female mice suppressed their inflammatory response while castration of male mice induced their susceptibility to acute cholangitis development (271). These data underscore the crucial role of testosterone in modulating immune responses in autoimmune liver disease. Diabetes induced by HFD/Streptozotocin (STZ, an antibiotic that destruct pancreatic islet β cell), was associated with increased Th17/IL-17 levels in the circulation and brain of female rats compared to males (272). However, the significance of such sexual dimorphic Th17 signature was not investigated. It is important to note here that a sex-specific difference in the Th17/IL-17 axis is also common among other diseases like ankylosing spondylitis (273), Sjögren’s syndrome (274), rheumatoid arthritis (275), and urinary tract infection (276). Taken together, studies across diverse diseases underscore the critical implication of sex hormones, particularly testosterone, in modulating Th17/IL-17 responses (**Figure 3**). This highlights the necessity for further investigation into the specific role of IL-17 in sex-specific immune dynamics in MASLD.

As to IL-22, most preclinical studies examining its role in MASLD were conducted exclusively in male mice, leaving knowledge gaps in the context of sex-based immunological differences in IL-22 between males and females (147, 232–234, 238, 239). Such an incomplete picture is also partly due to limited research regarding the endogenous hepatic IL-22 in human MASLD and the predominant focus on experimental studies. Our group has demonstrated elevated levels of hepatic IL-22 gene and protein expression in females compared to males in three different MASLD cohorts and *in vivo* models (277). This was associated with enrichment of hepatic Th17/IL-22, Th22, γδ T cells, and neutrophils as major IL-22 producers in females. Our study aligns with other reports that demonstrated reduced or unchanged levels of hepatic IL-22 expression in males, including humans and mice, with MASLD compared to healthy controls (232, 233, 278). Moreover, a similar signature of sexual dimorphism in IL-22 has been reported in the APAP-induced liver injury model, where females had increased expression of hepatic IL-22 levels compared to males (279). Notably, our results also showed that the endogenous expression of IL-22BP was elevated in livers of female mice and not males, with MASLD (277). This increased IL-22BP expression was not associated with impaired overall bioactivity of IL-22, highlighting a protective function of the IL-22BP itself in the context of MASLD. We further validated the sex-dependent influence of IL-22 in HFD-induced MASLD models and demonstrated that a deficiency of IL-22 receptor signaling (*Il22ra1^-/-^* mice) exacerbated liver injury, hepatocyte death, lobular inflammation, and the advancement of MASH-related fibrosis in female but not male mice (277). Therefore, our results support the hepatoprotective effects of the endogenous IL-22/IL-22RA1 axis against MASLD-related inflammation and fibrosis in a sex-dependent manner. This effect was mediated by IL-22-induced antiapoptotic (e.g., BCL2) and antioxidant signals (e.g., MT2), suppressing hepatocyte death (**Figures 2**, **3**).

These novel findings may suggest a potential role of the female sex hormones in mediating hepatoprotective effects through the regulation of IL-22 expression. Although this remains a hypothesis to be tested, it is supported by several observations. *In vitro*, the synthetic progestin, medroxyprogesterone acetate (MPA), increased IL-22 expression in human Th22 cell clones but did not affect IL-22 production by Th17 cells (280). Additionally, LPS- or anti-CD3/CD28-stimulated splenocytes of female mice produced low levels of IL-22 upon their treatment with testosterone or dihydrotestosterone as compared to cells from males (279). Oral contraceptives increased serum IL-22 levels and the IL-22/IL-22BP ratio in females with polycystic ovary syndrome compared to healthy controls (281). Research, on the other hand, lacks a clear explanation for the reduction of endogenous IL-22 serum or hepatic levels seen in male mice upon diet-induced MASLD or obesity challenge. Importantly, this phenomenon seems not to depend on the type of diet used or the duration of diet induced obesity or MASLD models (232, 233, 237, 277), suggesting that low hepatic IL-22 levels appear stable regardless of the severity of metabolic inflammation. There is extensive evidence of low testosterone levels observed specifically in males with obesity, MetS, and MASLD (282), however little data exist linking testosterone levels with the decreased hepatic IL-22 seen in these patients. Taken together, further studies are essential to unravel the hormonal regulatory mechanisms that contribute to the sex difference in IL-22 expression in MASLD.

## Conclusion and future perspective

The pivotal roles of type 3 cytokines, IL-17 and IL-22, emerge prominently in MASLD. Accumulating evidence points toward IL-17 contributing to persistent inflammation and the progression of MASLD-related fibrosis, while IL-22 exerts a hepatoprotective role, mitigating inflammation and fibrosis associated with the disease. Sex-based differences in hepatic endogenous IL-22 levels and its binding protein IL-22BP have been reported in MASLD and ALIs, while sexual dimorphism of IL-17 in these conditions is largely unexplored. The sexual dimorphism of IL-22 is associated with protective effects in females, ameliorating MASLD-related inflammation, and delaying fibrosis progression. Nevertheless, it remains unclear whether this sex-specific IL-22 signature is dependent on estrogen. Additionally, the influence of sex differences on gut microbiota and how it modulates immune responses like type 3 cytokines during MASLD progression is still lacking. This underscores the necessity for further experimental and clinical investigations to explore sexual dimorphism of IL-22 and IL-17, in the context of hormonal regulatory mechanisms, in MASLD. Exploring IL-22 and IL-17 as potential therapeutics for CLDs has shown promising outcomes in clinical trials. However, the efficacy and safety of targeting these pathways in human MASLD remain unknown and necessitate further in-depth investigation. Therapeutic approaches for MASLD treatment should be tested in both sexes and sexual dimorphism in inflammatory responses and disease progression should be considered.

## Author contributions

MA: Visualization, Writing – original draft, Writing – review & editing. GH: Writing – original draft, Writing – review & editing. NS: Funding acquisition, Supervision, Writing – review & editing.
